# Shared Plasma Fatty Acid Profiles in Four Cancer Types Enable Diagnosis and Discrimination of Gastrointestinal and Lung Cancers

**DOI:** 10.3390/metabo16020128

**Published:** 2026-02-12

**Authors:** Ahad Hussain, Kangwe Shen, Yan Yan, Xuejun Kang, Li Xie

**Affiliations:** 1 Key Laboratory of Environmental Medicine and Engineering, Ministry of Education, School of Public Health, Southeast University, Nanjing 210009, China; ahadhussain@aup.edu.pk (A.H.);; 2 Key Laboratory of Child Development and Learning Science of Ministry of Education of China, School of Biological Sciences and Medical Engineering, Southeast University, Nanjing 210096, China; 3 College of Animal Science and Technology, Jinling Institute of Technology, Nanjing 210038, China

**Keywords:** cancer patients, fatty acids, metabolic alterations, biomarkers, GC-MS

## Abstract

**Background:** Cancer is a leading cause of mortality worldwide, characterized by metabolic reprogramming, including alterations in fatty acid (FA) metabolism. Plasma FA profiles hold promise as non-invasive biomarkers for the diagnosis and classification of cancer. **Objectives:** This study aimed to investigate the diagnostic potential of plasma FA profiles across four major cancers and to identify shared and cancer-type-specific metabolic alterations. **Methods:** We examine comprehensive FA profiling of plasma samples from 368 individuals, including patients with colorectal (CRC, n = 94), gastric (GC, n = 55), esophageal (EC, n = 53), and lung cancer (LC, n = 73), alongside 93 healthy controls (HCs) by gas chromatography–mass spectrometry. Data were analyzed using univariate statistics and multivariate modeling analysis. **Results:** Univariate analysis showed a shared set of altered FAs across the cancer types, demonstrating a shared pan-cancer metabolic shift. A comprehensive comparison revealed a remarkable shared pattern within the gastrointestinal (GI) cancers (GC, CRC, EC), while LC showed opposite trends for most FAs. Partial Least Squares Discriminant Analysis (PLS-DA) models on a 70% training set excellently discriminated each cancer type from HCs. The cross-validation of the model demonstrated robust internal performance with Q^2^ = 0.675 (LC), 0.559 (GC), 0.774 (CRC), and 0.628 (EC). This is followed by assessing the diagnostic accuracy on a 30% hold-out test set, with area under the curve (AUC) values of 0.686 (LC), 0.926 (GC), 0.905 (CRC), and 0.843 (EC). **Conclusions:** Plasma FA profiles may provide a potential source of biomarkers, capturing both shared cancer markers and distinct tissue-specific metabolic alterations. These findings highlight the high diagnostic and classificatory potential of FAs alterations in oncology.

## 1. Introduction

Cancer is a significant public health issue and is the second leading cause of death worldwide [1]. According to estimations from GLOBOCAN 2022, there were 19,976,499 new cases (including non-melanoma skin cancer) diagnosed worldwide in 2022; nearly 24.2% of these cases (4,824,703) were reported in China, including 2,533,906 males and 2,290,797 females [2]. Various factors, such as demographic, ecological, genetic influences, and cultural environment, contribute to wide-ranging cancer incidence rates, mortality rates, and DALY burdens [3]. Although significant advancements have been made in early detection and treatment, cancer remains a complex and multifactorial disease that presents a significant challenge to global health [4]. The resistance of cancer to conventional therapies, its heterogeneity across different organs, and its ability to metastasize highlight the urgent need for novel diagnostic and therapeutic approaches to combat the problem [5]. In this context, a comprehensive understanding of the molecular mechanisms underlying the progression of cancer is critical to developing more effective treatments and improving patient outcomes.

A common hallmark of cancer cells is their metabolic reprogramming to sustain the production of ATP and macromolecules needed for the growth of the cell, survival, and division [6]. In particular, the significance of altered fatty acid metabolism in cancer has received renewed interest as, aside from their principal role of structural components of the membrane matrix, they are also critical secondary messengers and can serve as fuel sources for energy production [7]. More than 100 years of investigation have provided incredible insights into the essential role of fatty acids in tumorigenesis [7]. The composition of fatty acids is tightly regulated in normal cells, but in cancer cells, its metabolic rewiring often leads to significant alterations [8]. These alterations might not only reflect the reprogramming of metabolic processes in response to the rapid energy demands of tumor cells but also may influence vital tumor-related processes such as cell survival, proliferation, migration, and metastasis [9,10].

Understanding altered fatty acid profiles as potential biomarkers for cancer diagnosis, prognosis, and treatment response is growing. Targeting fatty acid profiles could provide new strategies for cancer treatment, particularly for tumors that exhibit resistance to conventional therapies [11]. However, the alteration pattern in fatty acid profiles in cancer patients seems inconsistent in different types of cancer. Therefore, this study aims to explore the significance of altered fatty acid profiles and dysregulated metabolic pathways in various types of cancer by gas chromatography–mass spectrometry (GC–MS) to highlight their implications as potential biomarkers and for clinical outcomes. We specifically focus on the common alterations of fatty acid profiles and metabolic pathways across the cancer types, including those of gastrointestinal system cancers and lung cancer (LC). The gastrointestinal cancers include gastric cancer (GC), colorectal cancer (CRC), and esophageal cancer (EC).

## 2. Materials and Methods

### 2.1. Samples

The samples were collected from patients with GC (n = 55), CRC (n = 94), EC (n = 53), and LC (n = 73). In addition, 93 healthy control (HC) subjects were recruited with no cancer markers or chronic disease. The control group matched the cancer groups in terms of age and gender. Due to the clinical sample collection’s nature, detailed information regarding the stages of cancer, treatment history, nutritional status, BMI, and fasting information was not available for the subjects. The cohort represents a real-world mixed population of cancer patients at different points in their clinical management. The plasma samples were stored at −20 °C in closed glass containers until analysis. The study was conducted in accordance with the Declaration of Helsinki and approved by the Ethical Research Committee of Southeast University. Prior permission was granted by the IRB of Affiliated Hospital of Nanjing University of Chinese Medicine (Jiangsu Province Hospital of Chinese Medicine) (2022NL-218-02).

### 2.2. Standards

The standard product contains a total of 37 different components, with each compound contributing approximately 2.63% to the overall mixture. Notably, C16:0 Methyl Palmitate was the most abundant, representing 5.26% of the mixture. This composition is in accordance with the GB/T 22223-2008 standard [12] (Chinese Standard GB/T 22223-2008, 25 July 2024). There was a total of 18 saturated fatty acids (SFAs), nine monounsaturated fatty acids (MUFAs), and 10 polyunsaturated fatty acids (PUFAs). The SFAs fatty acids included methyl butyrate (C4:0), methyl hexanoate (C6:0), methyl octanoate (C8:0), methyl decanoate (C10:0), methyl undecanoate (C11:0), methyl laurate (C12:0), methyl tridecanoate (C13:0), methyl myristate (C14:0), methyl pentadecanoate (C15:0), methyl palmitate (C16:0), methyl heptadecanoate (C17:0), methyl stearate (C18:0), methyl arachidate (C20:0), methyl heneicosanoate (C21:0), methyl behenate (C22:0), methyl tricosanoate (C23:0), methyl lignocerate (C24:0), methyl arachidate (C25:0). MUFA included methyl myristoleate (C14:1), methyl 10-pentadecenoate (C15:1) methyl palmitoleate (C16:1), methyl 10-heptadecenoate (C17:1), methyl oleate (C18:1), methyl elaidate (C18:1), methyl erucate (C22:1), methyl 11-heptacosenoate (C23:1), methyl nervonate (C24:1). PUFA included methyl linoleate (C18:2), methyl linolenate (C18:3), methyl arachidonate (C20:4), methyl eicosapentaenoate (C20:5), methyl docosahexaenoate (C20:6), methyl 11-14-17 eicosatrienoate (C22:3), methyl homogamma linolenate (C22:4), methyl docosahexaenoate (C22:6), methyl docosadienoate (C24:2), and methyl eicosapentaenoate (C24:5). Another product contains only short-chain fatty acids, such as acetic acid (C2:0), propionic acid (C3:0), butyric acid (C4:0), isobutyric acid (C4:0) valeric acid (C5:0), isoaleric acid (C5:0), caproic acid (C6:0), and caprylic acid (C7:0).

### 2.3. Chemicals

Fatty acid standards were purchased from Shanghai Yuanye Bio-Technology Co., Ltd., (Shanghai, China). Methyl tert-butyl ether (for Chromatography, 99.8%), Na_2_SO_4_, and sodium chloride from Shanghai Chemical Agents Institute (Shanghai, China), ethyl acetate from Shanghai Aladdin Bio-Technology Co., Ltd., (Shanghai, China), while hexane was obtained from Shanghai Macklin Chemical Industry Technology Co., Ltd., (Shanghai, China). Methanol (HPLC Gradient Grade, 99.9%) was obtained from Jiangsu Huaiyin Fine Chemical Research Institute (Huaian, China); double-distilled water was used for the experiments.

### 2.4. Apparatus and Instruments

A Thermo Scientific gas chromatography–mass spectrometry (GC-MS) model (TRACE1300/1300) equipped with a TSQ mass spectrometric detector (Thermo Fisher Scientific, Waltham, MA, USA) was used for this study (Thermo TriPlus RSH, firmware version 2.2.15036.1111, hardware version 2.2.2). The front Inlet (SSL) carrier pressure was 293.33 (actual), column flow, purge flow, and split flow were set as 2.00 mL/min, 5.00 mL/min, and 20.0 mL/min, respectively, with 240 °C temperature. MS transfer line and ion source temperatures were set at 250 °C and 300 °C, respectively. The GC oven temperature and time were set at 40 °C, and the equilibration time was 2.00 /min. The column (Thermo Fisher Scientific, Waltham, MA, USA) parameters were 100 m, 0.250 mm (i.d.), and 0.20 µm thickness. The samples were automatically injected (volume 1.0 µL).

### 2.5. Bligh and Dyer Extraction Method

The Bligh and Dyer extraction method was used with some changes, e.g., the Bligh and Dyer method consists of chloroform and methanol at a ratio of 2:1 (*v*/*v*) as the extraction solvent and a final volume of 20 times the volume of the sample. Then, they add water or a salt solution (e.g., 0.9% NaCl solution) to cause phase separation. We used ethyl acetate as the extraction solvent to reduce time, cost, and toxicity. The specific steps of the method are as follows: The plasma samples were thawed at 4 °C, 30 μL was taken and mixed with 600 μL of ethyl acetate (solvent), vortexed for 5 min at room temperature, then 2 mL of desiccated water was added, and the samples were shaken thoroughly. Samples were centrifuged at 2000 rpm for 5 min at 4 °C. The lower phase (organic) layer was transferred to a new tube and dried with nitrogen blow. A 1 mL volume of 2.5% sulfuric acid–methanol solution was added to the dried sample, sealed and mixed, and then reacted at 60 °C for 3 h. After the reaction, the samples were cooled at room temperature, 1 mL (1.5%) sodium chloride solution and 2 mL n-hexane were added, vortexed for 1 min, and centrifuged again at 2000 rpm for 5 min at 4 °C. The lower-phase (organic) layer was removed and dried under a stream of nitrogen at 40 C. The final dried samples were dissolved in 0.5 mL of n-hexane and injected into GC-MS (1.0 µL) for determination and detection.

### 2.6. Quantification of Short-Chain Fatty Acids

The analysis of SCFAs in plasma was carried out using a modified method based on our group’s previously established protocol [13]. Samples were pretreated using packed-fiber solid-phase extraction (SPE) with polystyrene/polypyrrole (PS/PPy) nanofibers (5 mg) as the sorbent. To activate the SPE column with nanofibers, 200 μL of methanol and 200 μL of water were added progressively before extraction. A 100 μL plasma sample was placed onto the conditioned SPE column. The sample was pulled through the sorbent under controlled pressure with a barometer solid-phase extractor (Dongqi Bio-technology Co., Ltd., Xi’an, China). After loading the sample, the target substances were eluted with 100 μL of an ethanol solution containing 0.01 mol/L hydrochloric acid. A second cleanup procedure was used to eliminate any remaining protein precipitate from the eluent. The eluate was passed through a micro-tip column containing 1 mg of polystyrene nanofibers. The filtrate was then collected and injected directly into GC-MS apparatus for detection. All other instrumental settings and experimental conditions were as described previously [13].

### 2.7. Statistical Analysis

Data is presented as mean ± SD and colored as red (decreased level) and blue (increased level) to indicate metabolic differences. Differences in metabolite levels between cancer and HC were assessed using Student’s *t*-tests, with *p*-values corrected for multiple comparisons using the Bonferroni method. For multivariate analysis (diagnostic modeling), the cohort for each type of cancer was randomly divided into a training set (70%) and an independent test set (30%). The training set was used to build a Partial Least Squares Discriminant Analysis (PLS-DA) model. Feature (significant metabolites) was selected based on Variable Importance in Projection (VIP) scores > 1.0. The predictive performance of model was calculated using 5-fold cross-validation on the training set, producing metrics such as accuracy, R^2^, and Q^2^. The final model was then applied once to the hold-out test set to compute the unbiased area under the receiver operating characteristic curve (AUC). Metabolic Pathway Analysis (MetPA) was conducted to explore the metabolic pathways associated with the fatty acids that played a significant role in the analysis. The hypergeometric test was applied for over-representation analysis. Potential confounding from analytical batch was assessed by principal component analysis (PCA) with samples colored by batch number The statistical analysis was performed by MetaboAnalyst software (0.6), and some figures were plotted by Origin Pro 2021.

## 3. Results

### 3.1. Characteristics of Study Participants

The dataset included 368 blood samples, comprising 275 cancer patients and 93 age- and gender-matched HCs. Among the cancer patients, the distribution was as follows: 53 with esophageal cancer (EC), 73 with lung cancer (LC), 94 with colorectal cancer (CRC), and 55 with gastric cancer (GC). The average ages of patients with esophageal, lung, breast, colorectal, and gastric cancer were 64.5 years (range: 39–80), 60.6 years (range: 35–78), 54.7 years (range: 26–70), 57.3 years (range: 24–75), and 58.6 years (range: 27–79), respectively. The demographic characteristics of the participants are summarized in Table 1.

### 3.2. Univariate Analysis

**Figure 1:**
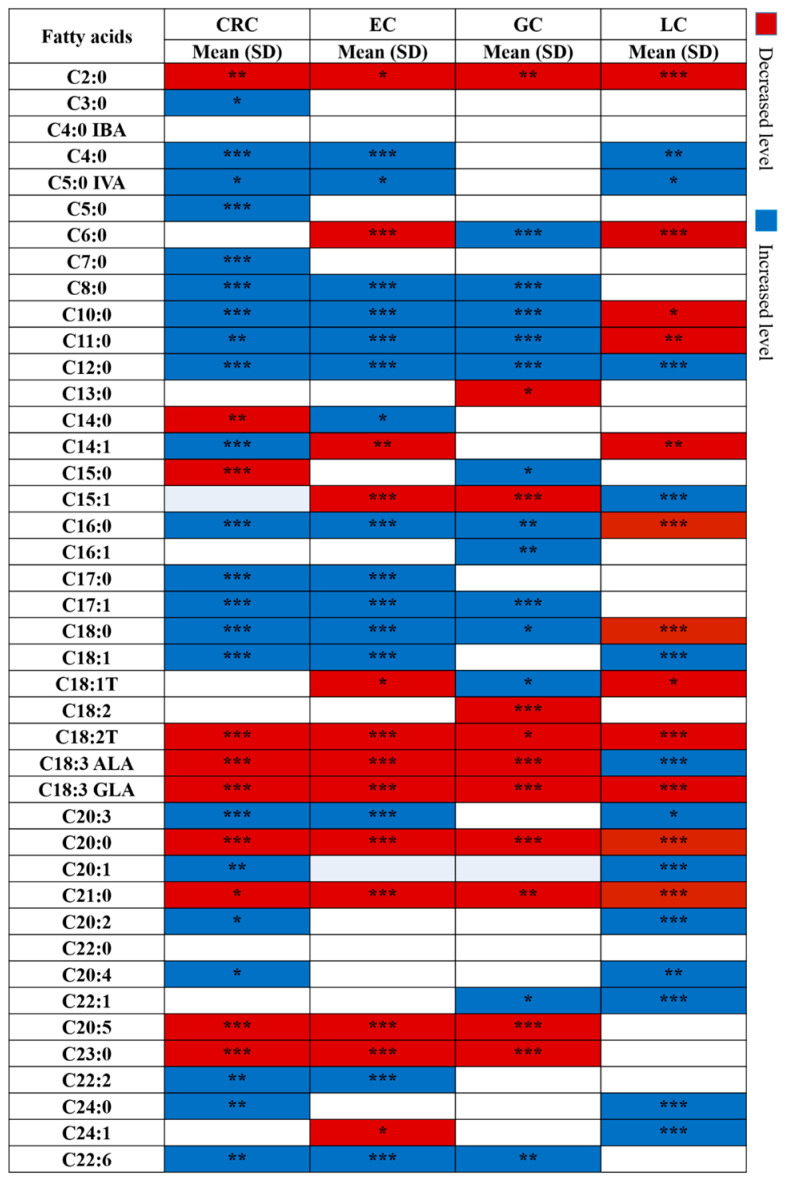
The differential fatty acids in all types of cancers. The red color indicates a significantly decreased level, while the blue color indicates an increased level with Bonferroni-adjusted *p*-value. *p* < 0.05. *** *p* < 0.001, ** *p* < 0.01, * *p* < 0.05.

Univariate analysis was performed to compare the fatty acid profiles of each type of cancer patient and HCs. The differences in the significance levels of each fatty acid between the cancer patients and the HC group are shown in Figure 1.

**Figure 2:**
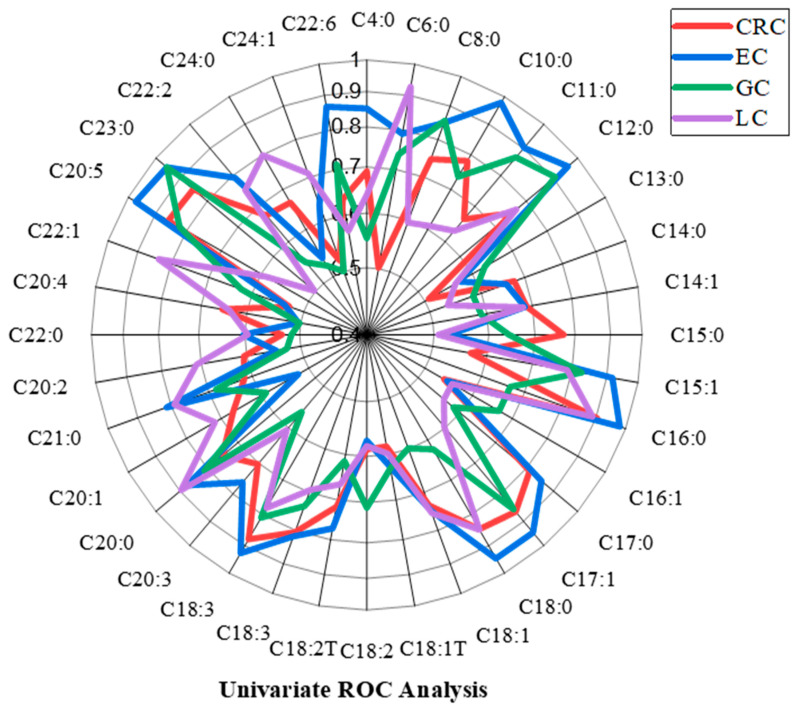
Univariate ROC analysis. The figure shows univariate ROC analysis for all the fatty acids across the cancer groups.

Red cells indicate significantly decreased levels of fatty acids for each cancer type, and blue cells indicate significantly increased levels. Univariate ROC analysis of all the fatty acids was performed to determine the effect of using each fatty acid singly to classify the subjects into cancer patients or HCs (Figure 2).

### 3.3. Common Alteration of Metabolites Across Cancer Types

The concentration of fatty acid profiles across the different types of cancer is summarized in Figure 1. Univariate analysis showed a core set of fatty acids that were commonly altered across the cancer types, indicating a shared pan-cancer metabolic shift. Specifically, levels of acetic acid (C2:0), linolelaidic acid (C18:2T, *trans*), γ-linolenic acid (GLA, C18:3n-6), arachidic acid (C20:0), and heneicosanoic acid (C21:0) were consistently decreased, while lauric acid (C12:0) was elevated across the cancers.

Beyond these common changes, a detailed comparison between gastrointestinal (GI) cancers (EC, GC, CRC) and LC showed that the majority of fatty acids exhibited strongly opposing trends. For example, in GI cancers, saturated fatty acids such as caprylic (C8:0), capric (C10:0), undecanoic (C11:0), palmitic (C16:0), heptadecanoic (C17:0), and stearic acid (C18:0), along with docosahexaenoic acid (DHA, C22:6n-3), were significantly increased. In contrast, LC showed the opposite trend for these same metabolites, showing either significant depletion or no change. This reciprocal pattern extended to key polyunsaturated fatty acids (PUFAs). In GI cancers, α-linolenic acid (ALA, C18:3n-3), eicosapentaenoic acid (EPA, C20:5n-3), tricosanoic acid (C23:0), and γ-linolenic acid (GLA, C18:3n-6) decreased. Similarly, LC showed the opposite trend for the same metabolites, showing either elevation or no change. Altogether, GI cancers display a lipid enrichment profile that is inversely mirrored in LC. Alterations in these metabolites might therefore reflect characteristic changes in metabolism that are common to all cancers, particularly GI cancers.

### 3.4. Specific Fatty Acid Profiles for Each Cancer Type

In addition to the changes in fatty acid profiles that were common to all cancers, we detected alterations in fatty acid profiles that were specific to each cancer type. Overall, the concentrations of most of the fatty acids showed an opposite trend between GI cancers and LC. However, some of the fatty acids showed opposite trends in each type of cancer. For example, the concentrations of C6:0 increased in LC while decreased in EC and GC, the concentration of C18:1T increased in GC while decreased in EC and LC, the concentration of C14:0 increased in EC and decreased in CRC, the concentration of C15:0 increased in GC while decreased in CRC, and C24:1 increased in LC and decreased in EC. These variations in the fatty acid profiles might reflect specific characteristics of each cancer and may serve as potential biomarkers for distinguishing cancer types. They warrant further exploration in the context of cancer metabolism.

### 3.5. Multivariate Analysis

In order to ensure model robustness and avoid overfitting, a stringent validation framework was employed for each type of cancer. The cohort for each type of cancer was split into training sets (70%) and hold-out test sets (30%). The training sets were used for model development and the independent test sets were used for final validation of the trained models. PLS-DA models were built on the training set. Features were selected based on whether VIP > 1 (Component 1); 5-fold cross-validation was performed to obtain internal performance estimates (accuracy, R^2^, Q^2^). Finally, the trained models (significant features from Component 1) were applied once to the hold-out test set for final validation.

#### 3.5.1. Colorectal Cancer (CRC)

**Figure 3:**
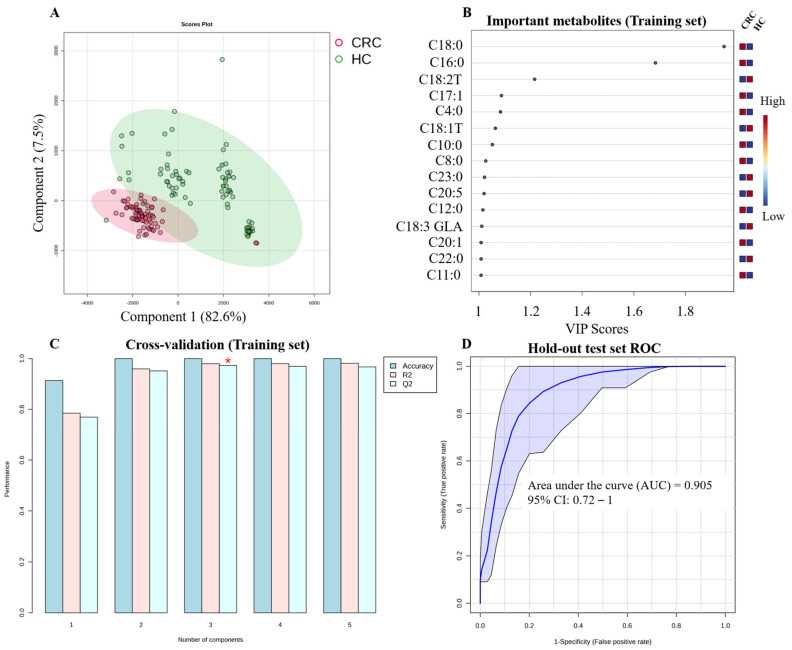
Diagnostic model for colorectal cancer (CRC). (**A**) PLS-DA score plot showing separation between CRC patients and healthy controls based on the 70% training set. (**B**) Variable Importance in Projection (VIP) plot of significant metabolites selected from the training set (VIP > 1.0). (**C**) Results of 5-fold cross-validation on the training set. (**D**) Receiver Operating Characteristic (ROC) curve for the final model validation, applied to the independent hold-out test set (30%). The red asterisk shows the highest Q2 values.

The PLS-DA score plot illustrates a separation between CRC and HC (Figure 3A). The training set identified a panel of discriminatory metabolites such as C18:0, C16:0, C18:2T, C17:1, C4:0, C18:1T, C10:0, C8:0, C23:0, C20:5, C12:0, C18:3 GLA, C20:1, C22:0, and C11:0, based on VIP > 1 (Figure 3B). The cross-validation of the model demonstrated robust internal performance with an accuracy = 0.96, R^2^ = 0.786, and Q^2^ = 0.774 (Figure 3C). The final model applied to the independent hold-out test set achieved an AUC = 0.905 confirming robust generalizability (Figure 3D).

#### 3.5.2. Esophageal Cancer (EC)

**Figure 4:**
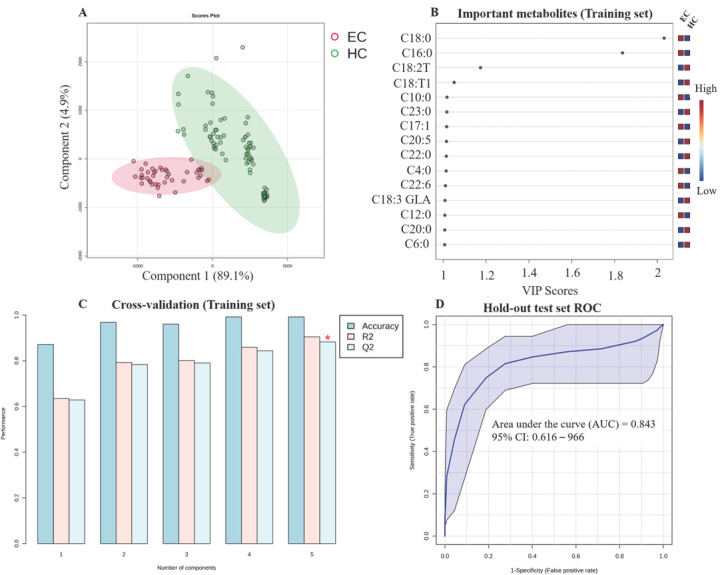
Diagnostic model for esophageal cancer (EC). (**A**) PLS-DA score plot showing separation between EC patients and healthy controls based on the 70% training set. (**B**) Variable Importance in Projection (VIP) plot of significant metabolites selected from the training set (VIP > 1.0). (**C**) Results of 5-fold cross-validation on the training set. (**D**) Receiver Operating Characteristic (ROC) curve for the final model validation, applied to the independent hold-out test set (30%). The red asterisk shows the highest Q2 values.

A similar analytical method was applied to EC. The PLS-DA model score plot showed distinct clustering of EC and HC (Figure 4A). Significant metabolites included in this model were C18:0, C16:0, C18:2T, C18:T1, C10:0, C23:0, C17:1, C20:5, C22:0, C4:0, C22:6, C18:3 GLA, C12:0, C20:0, and C6:0 (Figure 4B). The internal cross-validation yielded an accuracy = 0.87, R^2^ = 0.635 and Q^2^ = 0.628 (Figure 4C). The model was validated on the independent hold-out test set with an AUC = 0.843 (Figure 4D).

#### 3.5.3. Gastric Cancer (GC)

**Figure 5:**
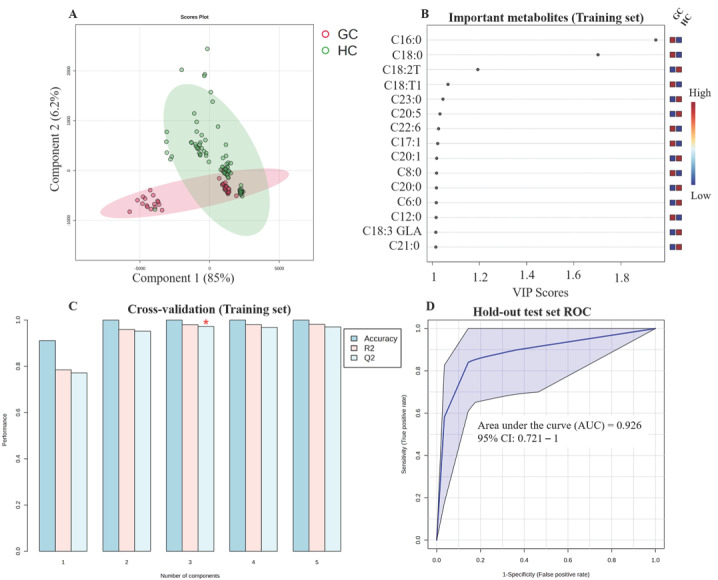
Diagnostic model for gastric cancer (GC). (**A**) PLS-DA score plot showing separation between GC patients and healthy controls based on the 70% training set. (**B**) Variable Importance in Projection (VIP) plot of significant metabolites selected from the training set (VIP > 1.0). (**C**) Results of 5-fold cross-validation on the training set. (**D**) Receiver Operating Characteristic (ROC) curve for the final model validation, applied to the independent hold-out test set (30%). The red asterisk shows the highest Q2 values.

For GC, the PLS-DA score plot illustrates separation between the group (Figure 5A). Significant metabolites included in the model were C16:0, C18:0, C18:2T, C18:T1, C23:0, C20:5, C22:6, C17:1, C20:1, C8:0, C20:0, C6:0, C12:0, C18:3 GLA, and C21:0 (Figure 5B). The model exhibited internal cross-validation with an accuracy = 0.819, R^2^ = 0.58, and Q^2^ = 0.559 0.83 (Figure 5C). The test set resulted in an AUC of 0.926 (Figure 5D).

#### 3.5.4. Lung Cancer (LC)

**Figure 6:**
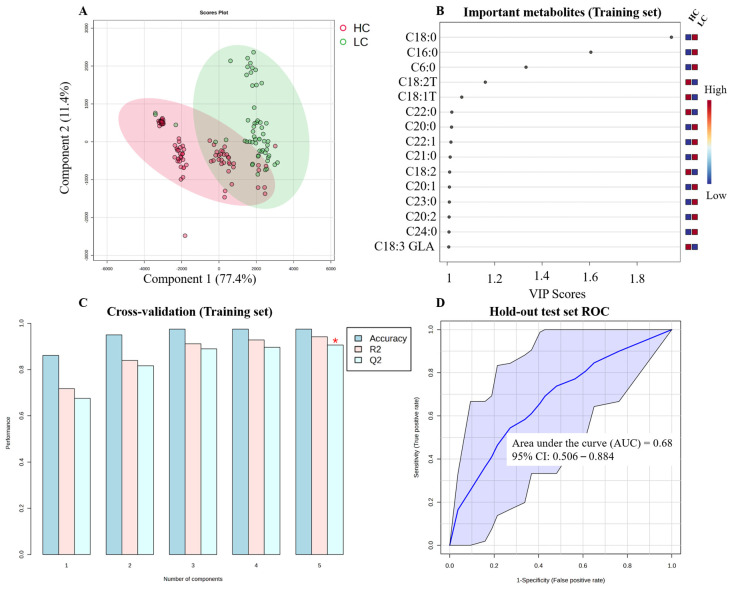
Diagnostic model for lung cancer (LC). (**A**) PLS-DA score plot showing separation between LC patients and healthy controls based on the 70% training set. (**B**) Variable Importance in Projection (VIP) plot of significant metabolites selected from the training set (VIP > 1.0). (**C**) Results of 5-fold cross-validation on the training set. (**D**) Receiver Operating Characteristic (ROC) curve for the final model validation, applied to the independent hold-out test set (30%). The red asterisk shows the highest Q2 values.

The LC model also showed significant discriminatory power, as shown in the PLS-DA visualization (Figure 6A). The discriminatory metabolites included in the model were C18:0, C16:0, C6:0, C18:2T, C18:1T, C22:0, C20:0, C22:1, C21:0, C18:2, C20:1, C23:0, C20:2, C24:0, and C18:3 GLA (Figure 6B). The internal cross-validation yielded an accuracy = 0.861, R^2^ = 0.717 and Q^2^ = 0.675 (Figure 6C). The hold-out test set confirmed its diagnostic effectiveness, with an AUC= 0.686 (Figure 6D).

The performance metrics for all the four cancer-specific models and the consistent pattern of high cross-validated metrics coupled with the strong independent hold-out test set highlight the robustness and generalizable diagnostic potential of plasma fatty acid profiling.

### 3.6. Distinguishing Cancer Types by PLS-DA

**Figure 7:**
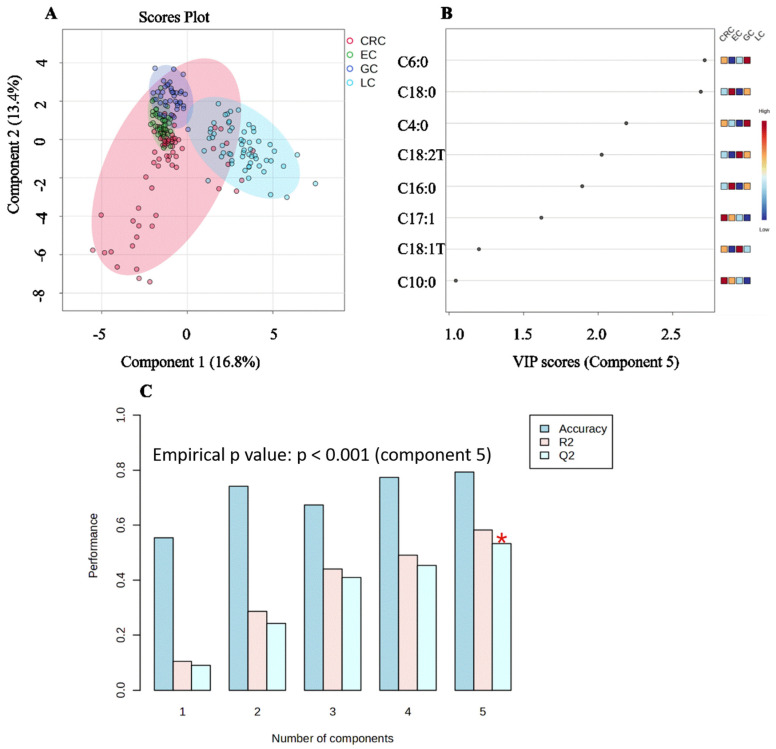
PLS-DA model discriminating cancer types. (**A**) Scores plot for the first two latent components (t[1] vs. t[2]) with 95% confidence ellipses. (**B**) Variable Importance in Projection (VIP) scores for metabolites selected in the component 5 model. (**C**) Model performance optimization. The component 5 model was selected as optimal, achieving a classification accuracy of 79.3% and a predictive Q^2^ of 0.533. The red asterisk shows the highest Q2 values.

The PLS-DA model showed discrimination among the cancer types. LC formed the most divergent cluster, showing slight overlap with the GI cancers, particularly EC and GC in the latent variable space (Figure 7A). The three GI cancers exhibited greater inter-cluster proximity, suggesting shared metabolic features despite their anatomical differences.

This obvious separation of LC may be the key driver of the model’s classification accuracy. The key discriminatory metabolites are presented in Figure 7B (VIP > 1), revealing that the VIP list may be dominated by the compounds whose concentrations were uniquely increased or decreased in LC relative to all GI cancers, which is consistent with the opposing trends observed in the univariate analysis.

The ideal complexity of the PLS-DA model for discriminating between the cancer types was determined through 10-fold cross-validation. The model performance was evaluated by classification accuracy and predictive metrics (R^2^ and Q^2^) and validated by a permutation test (1000), *p* < 0.001 (Figure 7C). Moreover, the incremental results with the addition of latent components are shown in Table 2.

The component 1 model performed poorly with an accuracy of 55.4% and negligible explanatory power (R^2^ = 0.10). The performance of the models improved with additional components, reaching a clear optimum at component 5. This final model (component 5) achieved a robust cross-validated classification accuracy of 79.3%, which is significantly higher than chance (25% for four groups). It explained a considerable proportion of the variance in the class labels (R^2^Y = 0.58), and most importantly, this model demonstrated a good predictive reliability, as indicated by a cross-validated predictive metric of Q^2^ = 0.53. According to established conventions in chemometrics, a Q^2^ value above 0.5 is considered good and indicates a model with valid predictive ability for biological data. Therefore, the component 5 PLS-DA model was selected for subsequent analyses, including the identification of discriminatory metabolites between four types of cancers.

### 3.7. Metabolic Pathway Analysis

**Figure 8:**
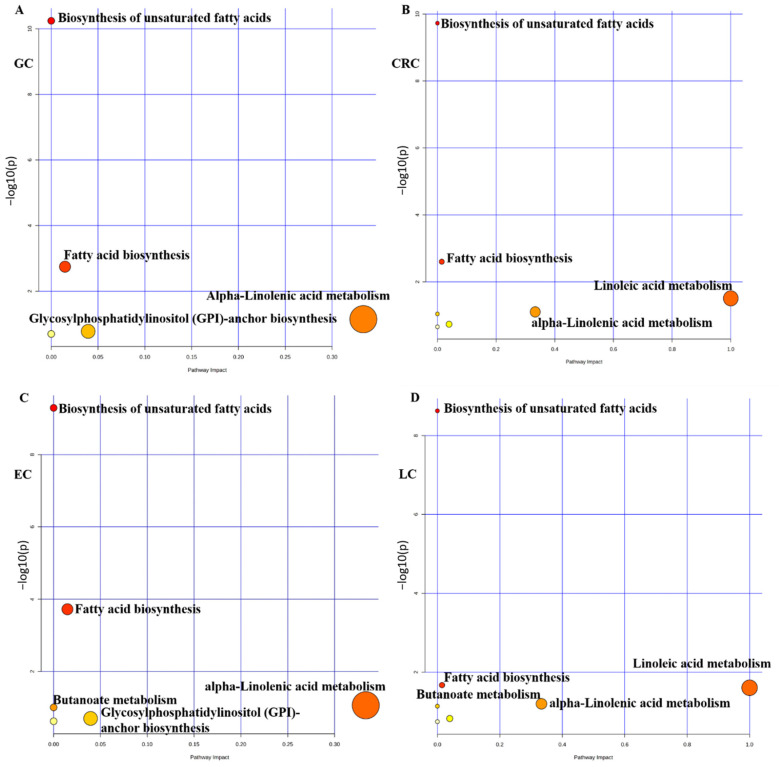
Metabolic pathway analysis, Metabolic pathway analysis of gastric cancer (**A**), colorectal cancer (**B**), esophageal cancer (**C**), and lung cancer (**D**). The size of the bubble represents the pathway impact or centrality, a larger bubble means the pathway is more central or influential. The color of the bubble represents the statistical significance. Red color typically represents the most significant level.

The significant metabolites included in the PLS-DA models (VIP > 1) for each cancer type were introduced in the pathways analysis module for metabolic pathway analysis (MetPA) in MetaboAnalyst software (version 6.0), https://www.metaboanalyst.ca. Hypergeometric test was selected for over-representation analysis. Commonly altered metabolic pathways were observed across the cancer groups, including fatty acid biosynthesis, biosynthesis of unsaturated fatty acids, and alpha-linolenic acid metabolism (Figure 8A–D).

## 4. Discussion

This study demonstrates that plasma fatty acid (FA) profiles captured two diverse yet informative layers of cancer-associated metabolic reprogramming. First, we identified a core set of commonly altered FAs across different types of cancer, and some findings were consistent with previous studies. For example, decreases in the concentrations of C20:5 [14], C18:3 ALA, C18:3GLA, and C18:2T [15], as well as increases in the levels of C16:0 and C18:0 [16] across cancer types, especially in GI cancers, reinforce the concept of a conserved pan-cancer metabolic shift necessary for sustained proliferation. More significantly, our results revealed a remarkable pattern of FA profiles that are shared across gastrointestinal (GI) cancers and distinguish the GI cancers from LC. The robust diagnostic performance of classifiers built on these signatures achieved excellent sensitivity and specificity and validates their translational potential. Furthermore, discussing each shared or specific fatty acid observed across the cancers may be complex and confusing. Therefore, we focused on the shared altered metabolic pathways across the cancers based on the metabolites included in multivariate models with VIP > 1. However, the univariate analysis findings are also discussed shortly.

The univariate analysis identified a core set of fatty acids consistently altered across the cancer types studied (EC, GC, CRC, and LC). These pan-cancer markers, including fatty acids such as common decreases in acetic acid (C2:0), linolelaidic acid (C18:2T), γ-linolenic acid (GLA, C18:3n-6), arachidic acid (C20:0), and heneicosanoic acid (C21:0), alongside an elevation in lauric acid (C12:0) across the four types of cancers, indicate shared alteration patterns that may point to fundamental metabolic perturbations inherent to the malignant state. Furthermore, the increase in C12:0 across the cancer groups should be considered cautiously. C12:0 is a key component of the widely used laboratory surfactant sodium lauryl sulfate, which is extremely vulnerable to environmental contamination. Because procedural blanks were not examined in our study, we therefore cannot rule out technical artifacts as the cause of this signal. As a result, we refrain from assigning biological relevance to the C12:0 discovery and emphasize that future investigations need to include blank controls to confirm its origin.

The decreased level of C2:0 in all types of cancers may reflect a common disruption in host–microbiome metabolic crosstalk or a shift away from acetate utilization for acetyl-CoA pools [17,18]. Previous studies indicated that C2:0 is a potent anticancer agent, and a topical application of C2:0 may be a possible approach for the treatment of different types of cancers [19]. Therefore, in this study, this common reduction in C2:0 may be used as a key biomarker of cancer diagnosis. Further longitudinal studies are needed to validate our findings.

The reduction in specific unsaturated (C18:2T, GLA) and long-chain saturated (C20:0, C21:0) fatty acids in this study suggests a shared reprogramming of Stearoyl-CoA Desaturase-1 activity across the cancers and very-long-chain fatty acid synthesis, possibly to reduce the production of pro-apoptotic lipid species or to remodel membrane properties for enhanced survival signaling [20,21,22,23]. Conversely, increases in the medium-chain saturated fatty acid such as lauric acid may indicate a shared dependence on specific lipid species for energy or signaling, a hypothesis requiring further functional validation. While previous in vitro studies have recognized that exogenous lauric acid triggers a defined pro-apoptotic pathway in cancer cells [24], our finding of elevated endogenous lauric acid levels in four different cancer types presents a significant paradox. These findings suggest that within the innate tumor microenvironment, lauric acid’s role might be complex and context-dependent, which may potentially act as a marker of dysregulated lipid metabolism, a factor whose apoptotic function is circumvented in vivo or a tolerated stress signal. Our results shift the perspective on lauric acid from a solely therapeutic fatty acid to a related oncometabolite, highlighting a need to investigate further the functional consequences of its endogenous increase in cancer progression and survival.

The most compelling finding in this study is the opposing fatty acid profiles observed between GI cancers (EC, GC, CRC) and LC. For a majority of fatty acids, the direction of change was commonly reversed. For example, GI cancers showed a significant elevation in a broad spectrum of saturated fatty acids, such as those from medium-chain (C8:0, C10:0, C11:0) to long-chain fatty acids (C16:0, C17:0, C18:0), as well as from docosahexaenoic acid (DHA, C22:6n-3). In contrast, LC exhibited a reduction or non-significant changes in the same metabolites. In particular, the decreased level of C16:0 and C18:0 in CL patients contradicts the well-known “lipogenic phenotype,” in which tumors upregulate FASN to synthesis these fatty acids. The most straightforward explanation for this systemic depletion may cancer-associated cachexia, (a common wasting disease in LC) that involves the mobilization and oxidation of adipose tissue storage, which depletes circulating lipid pools. Because BMI and weight loss histories were not recorded in our study, we cannot rule out cachexia as the underlying cause of this characteristic. An alternate, non-exclusive idea is that LC may cause a metabolic state that may favors fatty acid oxidation over storage.

Alpha-linolenic acid (ALA, C18:3n-3) and eicosapentaenoic acid (EPA, C20:5n-3) were reduced in GI cancers, while LC exhibited no significant change in EPA. A recent study reported that omega-3 PUFAs are immuno-enhancing. These fatty acids neutralize the immune-suppressive state of GI cancers by enhancing critical immune cells (T cells and CD4+ T cells) [25]. Our findings provide the metabolic evidence that these (immuno-enhancing omega-3) fatty acids were specifically depleted in GI cancers (but not in LC). This creates a dominant connection, suggesting that the tumor microenvironment in GI cancers may seem to be characterized by a selective loss of key immune-supportive lipids. The depletion in these fatty acids we observed may not be a neutral metabolic change, it probably may contribute directly to the immune-suppressive state described in the previous study [25]. By decreasing the local availability of these fatty acids, GI cancers may be creating a metabolic condition that further dampens anti-tumor immunity, facilitating tumor progression. Furthermore, the consistent reduction in the very-long-chain saturated fatty acid tricosanoic acid (C23:0) across all GI cancers (a change also absent in LC) emerges as a novel and unique metabolic hallmark of GI cancers, which has been limitedly reported previously. The functional significance of this fatty acid may be an important avenue for future research.

This “mirror-image” metabolic alteration suggests that GI cancers and LC fundamentally oppose lipid acquisition and utilization strategies. This variance may reveal the different metabolic needs of GI cancers compared to LC, as previous studies suggest that LC tends to favor oxidative phosphorylation and amino acid metabolism [9]. In contrast, GI cancers involve extensive lipid metabolic changes driven by inflammation and a higher rate of lipid synthesis [26]. The observed metabolic changes in fatty acid profiles may not only reflect tissue-specific reprogramming but may also provide valuable insights into the unique biological features of GI cancers and LC. These changes might potentially contribute to cancer progression and may offer new opportunities for distinguishing between different cancer types.

To rule out the potential that the observed “mirror-image” metabolic patterns between GI and LC may be due to technical anomalies (e.g., batch effects or run-date variability). Therefore, we applied principal component analysis (PCA) colored by batch number. Samples were processed in 11 analytical batches, with LC samples divided into three batches (Batches 6–8). As illustrated in Supplementary Figure S1, samples from these LC-associated batches did not form separate batch-specific clusters. Instead, some samples were mixed together with samples from GI tumors and healthy controls processed in separate batches. This suggests that the analytical batch did not dominate the major sources of variance in our dataset, reinforcing the conclusion that the observed fatty acid profile separation may be due to biological differences between the cancer types.

### 4.1. Metabolic Pathway Analysis

Furthermore, a set of metabolites observed in multivariate analysis (VIP > 1) in each cancer type was introduced to metabolic pathway analysis. The most significant commonly dysregulated metabolic pathways observed in each type of cancer were alpha-linolenic acid metabolism, biosynthesis of unsaturated fatty acids, and fatty acid biosynthesis (Figure 8A–D).

### 4.2. Alpha-Linolenic Acid Metabolism

Alpha-linolenic acid (C18:3-ALA) metabolism is an essential metabolic pathway in humans [27]. Meanwhile, the classic metabolic pathway might also significantly affect the biological behavior of cancer [28]. Wang et al. reported that ALA can suppress the migration of human triple-negative breast cancer cells by reducing Twist1 expression and suppressing Twist1-mediated epithelial–mesenchymal transition (EMT) [29], suggesting a potential mechanism for ALA’s anti-metastatic effects. Li et al. reported that dietary supplementation of ALA induced conversion of n-3 LCPUFAs in the mouse model and reduced prostate cancer growth [30]. However, there is limited research focused on the shared role of ALA metabolism in different types of cancers. It is vital to highlight that ALA is an essential fatty acid available primarily through diet. As a result, its observed depletion in GI cancers may most likely due to a combination of reduced dietary intake (anorexia, dysphagia) or intestinal malabsorption, that is, common complications of GI cancers [31], rather than a primary dysregulation of its enzymatic conversion pathway (e.g., via FADS1/2 activity). While this does not limit its potential as a nutritional status biomarker or its contribution to a disease-associated metabolic signature, it warns against misinterpreting the results as proof of specific enzymatic rewiring. Future research evaluating downstream elongation products (EPA, DHA) and precursor ratios in controlled nutritional contexts will help to distinguish between dietary and enzymatic impacts.

### 4.3. Biosynthesis of Unsaturated Fatty Acids

Another pathway that was significantly dysregulated across the cancer types was the biosynthesis of unsaturated fatty acids. Previous studies demonstrated that biosynthesis of unsaturated fatty acids is strongly associated with some critical hallmarks of cancer, such as the peroxisome pathway and the mTOR complex 1 pathway [32]. Particularly in the peroxisome pathway, Peroxisome Proliferator-Activated Receptor γ (PPARγ) is a nuclear receptor involved in regulating lipid metabolism-related gene expression, particularly PUFAs, which are the natural ligands of this receptor [33,34]. A study indicated that PUFA, such as those available in fish oil, can activate PPARγ, and their consumption is related to the prevention of colon cancer [35]. Our findings of dysregulated unsaturated fatty acid biosynthesis as a shared metabolic feature observed across the cancer types might provide a pan-cancer context for recognizing its role in PPARγ-mediated tumor suppression. This consistent alteration may suggest that tumors may commonly rewire this metabolic pathway to avoid the anti-proliferative signals associated with the PUFA-activated PPARγ. Targeting this dysregulated biosynthesis to restore endogenous PUFA levels might therefore represent a novel metabolic strategy to reactivate PPARγ-mediated tumor suppression across multiple cancer types.

### 4.4. Biosynthesis of Fatty Acids

Several studies have confirmed the importance of the biosynthesis of fatty acids for cancer cell growth and survival [36,37,38]. Fatty acids are substrates that produce lipid signaling molecules, and the dysregulation of fatty acid biosynthesis is closely associated with the growth and differentiation of cancer cells [39]. Expression of FASN is observed in proliferating fetal tissues [40], suggesting that reactivation of FA synthesis in cancer cells may represent a reversion to a less-differentiated embryonic state. Alternatively, increased fatty acid biosynthesis may be a response to the high metabolic demands of cancer cells or an adaptation to reduce the availability of serum-derived lipids in the tumor microenvironment. Recent evidence also proposes that genomic alterations, such as the deletion of chromosome 8p in breast cancer, activate FA synthesis [41], which indicates that FA synthesis may be crucial for cancer development and progression.

Furthermore, the metabolic intermediate that provides the substrate for the synthesis of FA is produced via several mechanisms. For example, acetyl-CoA carboxylases (ACCs) are enzymes that catalyze the carboxylation of acetyl-CoA to produce malonyl-Co. This malonyl-CoA then serves as the essential building block for fatty acid synthase (FASN) to produce long-chain, saturated fatty acids, such as palmitate (C 16;0) [36]. ATP-citrate lyase (ACLY) produces acetyl-CoA for the synthesis of fatty acids by converting citrate derived from the TCA cycle [7]. Sterol Regulatory Element-Binding Proteins (SREBPs), transcription factors, also regulate the expression of fatty acid synthesis genes, including FASN, and are activated by growth signals such as AKT [42]. Moreover, SCD1 converts saturated fatty acids into MUFAs that influence membrane fluidity and cell survival [43]. Lipin 1 (LPIN1) is involved in the SREBP regulation activity, which controls lipid biosynthesis [44]. Collectively, these enzymes and their regulatory mechanisms could be dysregulated in cancer, contributing to the increased demand for lipids that are vital for the biosynthesis of membranes and energy storage in rapidly proliferating tumor cells. Therefore, dysregulated biosynthesis of fatty acids may be a key metabolic feature that supports tumor growth and progression, making these enzymes potential targets for therapeutic strategies in cancer treatment.

Furthermore, while our findings clearly show a ’mirror-image’ depletion of certain fatty acids in LC patients compared to GI cancers, the biological source of this signature remains unexplored and is hypotheses generating. It is possible that this profile may reflect the increased incidence of cancer-related cachexia and metabolic wasting in advanced LC rather than a tumor-cell intrinsic metabolic program. Further studies incorporating longitudinal weight loss, BMI, and body composition data are needed to distinguish the contributions of systemic host response from true tumor-specific metabolism.

### 4.5. Study Limitations

The interpretation of our findings must take into account several limitations inherent in our study. First, because there were no clear clinical staging of cancer and treatment data, the identified signatures could be affected by disease progression or medication. Second, we were unable to account for important metabolic variables such as dietary habits, nutritional status, BMI, and comorbidities, all of which might affect plasma metabolite levels. Third, samples were not obtained under uniform fasting conditions that may result in physiological variance. While our thorough matching for age and gender reduces some bias, unmeasured variables may likely contribute to the observed metabolic differences. As a result, the diagnostic panels described here provide robust proof-of-concept evidence of cancer-related metabolic changes. Future prospective studies with rigorous pre-analytical standardization and detailed clinical phenotyping are needed to validate their eventual clinical usefulness, whether directly tumor-derived or due to systemic host alterations.

## 5. Conclusions

Our findings revealed that, despite different tissue-specific alterations, particularly the opposing metabolite profiles between gastrointestinal cancers (esophageal, gastric, and colorectal cancer) and lung cancer, dysregulation of three core metabolic pathways, such as alpha-linolenic acid metabolism, biosynthesis of unsaturated fatty acids, and fatty acid biosynthesis, was observed across the four cancer types. This may suggest a fundamental pan-cancer metabolic reprogramming where tissue-specific adaptations exist within a shared framework of lipid pathway hijacking. Targeting these shared pathways may offer promising strategies for broad therapeutic intervention, while the tissue-specific metabolic signatures may provide a basis for refining diagnostic and personalized treatment approaches. Furthermore, we highlight that these findings are hypothesis-generating, the lung cancer signature may mostly reflect cancer cachexia, and the origin of some markers (e.g., lauric acid) may remain uncertain without BMI data and process blanks, respectively. Thus, while these indicators offer high diagnostic and therapeutic potential, further prospective validation in cohorts with extensive clinical phenotyping to separate tumor-specific biology from systemic host responses and pre-analytical anomalies are needed.

## Figures and Tables

**Table 1:** Demographic characteristics of study participants.

Characteristics		Esophageal Cancer	Lung Cancer	Colorectal Cancer	Gastric Cancer	Healthy Control
No		53	73	94	55	93
Sex	Male	40	49	52	32	57
	Female	13	24	42	23	36
Age (years)	Mean	64.5	60.6	57.3	58.6	57.8

**Table 2:** Cross-validation performance metrics for the PLS-DA model across components.

Number of Components	Accuracy	R^2^Y	Q^2^
1	0.554	0.104	0.090
2	0.741	0.286	0.243
3	0.673	0.441	0.410
4	0.773	0.491	0.454
5	0.793	0.581	0.533

## Data Availability

The data are available from the corresponding author upon reasonable request.

## Supplementary Materials

The following supporting information can be downloaded at https://www.mdpi.com/article/10.3390/metabo16020128/s1, Figure S1: title.Principal component analysis (PCA) of all types of cancer samples colored by analytical batch number. Samples were processed in 11 analytical batches, lung cancer samples (analyzed in Batches 6, 7, and 8) are highlighted. The lack of clustering by batch numbers indicates that the technical variation does not explain the major metabolic differences observed between cancer groups, supporting a biological origin for the “mirror-image” lipid profiles.
