# Supplementary material for: Shared Plasma Fatty Acid Profiles in Four Cancer Types Enable Diagnosis and Discrimination of Gastrointestinal and Lung Cancers
Source: Metabolites. 2026 Feb 12;16(2):128. doi: 10.3390/metabo16020128 (PMC12943763; doi:10.3390/metabo16020128)
Supplement: Supplementary file 1 [file metabolites-16-00128-s001.zip › metabolites-4095496-supplementary.pdf]

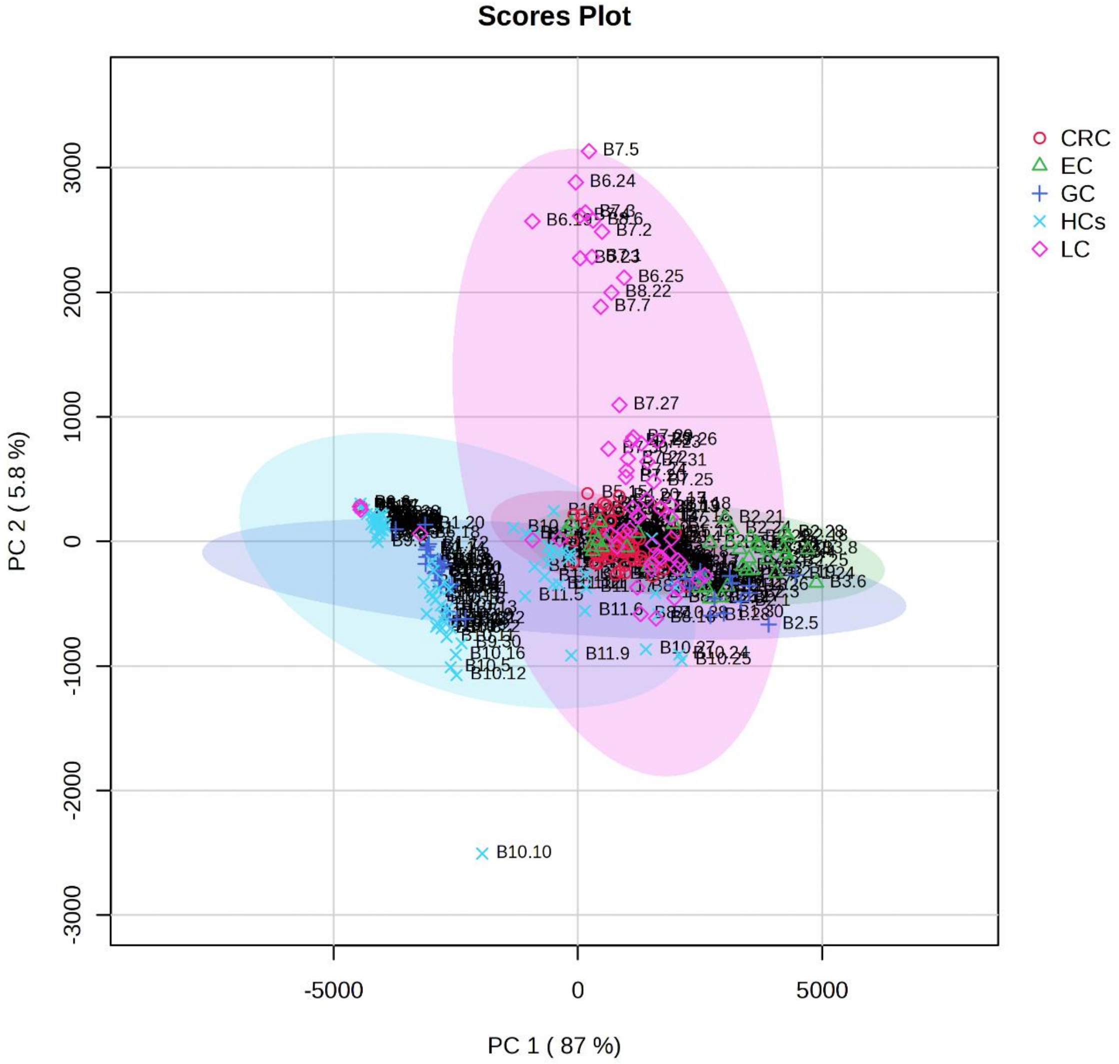

Supplementary Figure S1: Principal component analysis (PCA) of all type of cancers samples colored by analytical batch number. Samples were processed in 11 analytical batches, lung cancer samples (analyzed in Batches 6, 7, and 8) are highlighted. The lack of clustering by batch numbers indicates that the technical variation does not explain the major metabolic differences observed between cancer groups that supporting a biological origin for the "mirror-image" lipid profiles.
